# *White stripe leaf 12* (*WSL12*), encoding a nucleoside diphosphate kinase 2 (OsNDPK2), regulates chloroplast development and abiotic stress response in rice (*Oryza sativa* L.)

**DOI:** 10.1007/s11032-016-0479-6

**Published:** 2016-04-29

**Authors:** Weijun Ye, Shikai Hu, Liwen Wu, Changwei Ge, Yongtao Cui, Ping Chen, Xiaoqi Wang, Jie Xu, Deyong Ren, Guojun Dong, Qian Qian, Longbiao Guo

**Affiliations:** State Key Laboratory of Rice Biology, China National Rice Research Institute, Chinese Academy of Agricultural Sciences, Tiyuchang Road 359, Hangzhou, 310006 China; College of Agriculture and Biotechnology, Zhejiang University, Hangzhou, 310058 China; Agricultural Genomics Institute, Chinese Academy of Agricultural Sciences, Shenzhen, 518120 China

**Keywords:** Rice, WSL12, Chloroplast development, Abiotic stress, Map-based cloning

## Abstract

Chloroplast is a crucial organelle for plant photosynthesis and maintaining normal life activities in higher plants. Although some genes related to chloroplast development and pigment synthesis have been identified or cloned in rice, little is known about the relationship between these genes and abiotic stress response. In this study, we identified a novel mutant *white stripe leaf 12* (*wsl12*) affecting pigment synthesis, chloroplast development and abiotic stress response in rice. The mutant phenotype was obvious at seeding and tillering stages and in response to the temperature change. Genetic analysis of reciprocal crosses between *wsl12* and wild-type plants showed that *wsl12* was a recessive mutant in a single nuclear locus. Map-based cloning revealed that the *WSL12* locus encoded OsNDPK2, one of the three nucleoside diphosphate kinases (OsNDPKs). *WSL12* expressed in all tested tissues, while it highly expressed in leaves and young tissues. The WSL12 protein localized to the chloroplast. The *wsl12* mutant showed higher superoxide anion level and enhanced sensitivity to abscisic acid (ABA) and salinity. The transcription pattern of many genes involved in chlorophyll biosynthesis, ABA synthesis, light signaling pathway, reactive oxygen species-scavenging pathway and the other two *OsNDPKs* was altered in the *wsl12* mutant. These results indicate that the OsNDPK2 encoded by *WSL12* plays an important role in chloroplast development and chlorophyll biosynthesis by regulating the expression levels of related genes. In addition, *WSL12* also affects the response to abiotic stress, such as ABA and salinity in rice, and is beneficial to molecular breeding of stress tolerance.

**Electronic supplementary material:**

The online version of this article (doi:10.1007/s11032-016-0479-6) contains supplementary material, which is available to authorized users.

## Introduction

The transformation from proplastids to photosynthetically active chloroplasts is regulated by plastid and nuclear genes. The chloroplast proteins essential for chloroplast development and functions are mainly encoded by nuclear genes (Chen et al. 2010). Chloroplast only encodes about 100 genes because it is a semiautonomous organelle (Delannoy et al. 2009). The mutation of these genes may result in the generation of many mutants with visible leaf discoloration and abnormal seedling viability (Zhen et al. 2014; Guo et al. 2015). The mutants with abnormal pigment contents or deficient in chloroplast development are ideal materials for studying the underlying mechanisms that regulate chlorophyll biosynthesis and chloroplast development in rice. Many of these genes have been identified, including *OsCHLH*, *Chlorina 1*, *Chlorina 9*, *V1*, *V2*, *V3*, *ST1*, *OsPPR1* and *WSL* (Jung et al. 2003; Zhang et al. 2006; Kusumi et al. 2011; Sugimoto et al. 2007; Yoo et al. 2009; Gothandam et al. 2005; Tan et al. 2014).

Nucleoside diphosphate kinases (NDPKs) are highly conserved enzymes that catalyze the transfer of terminal phosphoryl group from nucleoside triphosphate (NTP) to nucleoside diphosphate through a ping-pong mechanism (Parks and Agarwal 1973). In animals, NDPKs participate in cell proliferation, differentiation, invasion and motility (Keim et al. 1992; Kantor et al. 1993). In plants, the main function of NDPKs is to maintain the balance between cellular NTPs and nucleoside diphosphates and provide NTPs for biosynthesis except for adenosine triphosphate (Roberts et al. 1997; Bernard et al. 2000). In addition to the housekeeping role, previous researches demonstrate that NDPKs are involved in other processes, such as phytochrome-mediated light signaling (Choi et al. 1999), UV-B signaling (Zimmermann et al. 1999), wounding (Harris et al. 1994), heat-shock response (Escobar Galvis et al. 2001), H_2_O_2_-mediated MAPK signaling (Moon et al. 2003) and auxin-mediated response (Choi et al. 2005). Plant NDPKs have been classified into three distinct types (type I–III) based on their amino acid sequences. All NDPKs sequences share a common catalytic histidine residue, and several of them exhibit an N-terminal extension for organellar targeting (Lascu and Gonin 2000; Dorion and Rivoal 2015). AtNDPK2 is considered to be linked with chloroplast function, oxidative stress and auxin signaling (Dorion and Rivoal 2015). Higher expression level of *AtNDPK2* is found in leaves and inflorescences, but lower or absent in roots (Verslues et al. 2007; Hammargren et al. 2007). Previous studies have shown that AtNDPK2 activity can be increased by binding to phytochrome A (Choi et al. 1999; Shen et al. 2005). The *Atndpk2* mutant showed deficient in cotyledon opening and greening in both red and far-red lights (Choi et al. 1999). These results indicate that *AtNDPK2* participates in the photomorphogenesis of leaves. NDPK2 can also regulate auxin-mediated responses for plant growth and development mainly through changing the expression of auxin-related genes to affect auxin transport (Choi et al. 2005). Among the three types of NDPKs, only NDPK2 is considered associated with reactive oxygen species (ROS) signaling and oxidative stress management. The *Atndpk2* mutant has higher ROS and H_2_O_2_ levels compared to its wild-type plants (Moon et al. 2003; Verslues et al. 2007). Under stress conditions, AtNDPK2 is involved in oxidative stress signaling by interacting with AtMPK3 and AtMPK6, two H_2_O_2_-activated *Arabidopsis thaliana* mitogen-activated protein kinases (Moon et al. 2003). *AtNDPK2* also participates in salt stress signaling by interacting with class 3 sucrose nonfermenting 1-related kinase (SOS2) and catalase (CAT; Verslues et al. 2007). *AtNDPK2* can regulate the activity of G protein since AtNDPK2 directly interacts with small G-proteins and controls their activities. It has been proposed that NDPK2 may be a missing link between the phytochrome-mediated light signaling and G protein-mediated signaling (Shen et al. 2008).

Many studies related to NDPK2 have been carried out in the dicotyledon model plant, and both the structure and function of AtNDPK2 have been well characterized in previous studies (Dorion and Rivoal 2015). However, little is known about the function of NDPK2 in rice (*Oryza sativa* L.). In this study, we investigated a *white stripe leaf 12* (*wsl12*) mutant, which exhibits white stripe leaf at seedling and tillering stages, and the phenotype is also affected by temperature. Genetic analysis of reciprocal crosses between *wsl12* and wild-type plants showed that *wsl12* was a recessive mutant in a single nuclear locus. Map-based cloning revealed that *WSL12* encodes the nucleoside diphosphate kinase 2 (OsNDPK2). Further researches demonstrate that *OsNDPK2* plays an important role in chlorophyll biosynthesis, chloroplast development and abiotic stress response. In addition, the transcription levels of the genes associated with abscisic acid (ABA) synthesis, ROS-scavenging pathway and light signaling pathway were changed in the *wsl12* mutant, which suggests the function of *WSL12* is pleiotropic.

## Materials and methods

### Plant materials and growing conditions

The rice *wsl12* mutant was obtained from the mutagenized populations of the *japonica* rice cultivar Wuyujing7 treated by EMS solution. The *wsl12* was crossed with the wild-type Wuyujing7 for genetic analysis. For fine mapping *WSL12* locus, a F_2_ population derived from the cross between *wsl12* and the *indica* cultivar 93-11 was used. Rice plants were grown in the paddy fields in Hangzhou (30°N latitude, summer season) and Sanya (18°N latitude, winter season) or in plant incubators (Panasonic, MLR-352H-PC). In the temperature experiments, seedlings were grown in incubators (12-/12-h light/dark; light intensity 300 μmol m^−2^ s^−1^) at the constant temperature of 22, 26, 30 and 34 °C conditions, respectively.

### Pigment content measurement

Chlorophyll and carotenoid concentrations were detected according to the method described by Arnon (1949) and Wellburn (1994). Leaf samples (0.15 g, fresh weight) were cut into segments, immersed in 10 ml 80 % acetone and incubated at 26 °C in dark for 24 h. The optical density of sample solutions was measured by ultraviolet spectrophotometer (DU800, Beckman, USA) with 663 nm (the maximum absorption peak of chlorophyll a), 645 nm (the maximum absorption peak of chlorophyll b) and 470 nm (the maximum absorption peak of carotenoid). Each sample was measured by three biological repeats.

### Transmission electron microscopy analysis of chloroplast ultrastructure

The leaves from the *wsl12* mutant and wild-type plants were cut into small pieces, placed in a phosphate buffer containing 2.5 % glutaraldehyde (pH = 7.2) and then vacuumed in a vacuum pumping machine until the leaves sank. The transmission electron microscopy samples were treated according to the method described by Gothandam et al. (2005). The processed samples were examined with a transmission electron microscope (Hitachi H-7650, Tokyo, Japan).

### NBT staining

Superoxide anion was detected by NBT staining according to the method described by Thordal-Christensen et al. (1997). The fully-expanded third leaves from *wsl12* mutant and wild-type plants were detached and soaked in NBT solution (0.5 mg/ml, NBT powder was dissolved with K_3_PHO_4_ solution, pH = 7.6) in the dark for 5 h.

### Genetic analysis and map-based cloning of *WSL12*

A total of 118 polymorphic markers scattered across all rice chromosomes and a DNA bulk pool from 33 individuals with the mutant phenotype of F_2_ population were used for preliminary linkage analysis. Subsequently, the fine mapping of *WSL12* was performed using 2257 plants with recessive phenotype. Simple sequence repeat markers were obtained from the Gramene database (http://www.Gramene.org). New polymorphic markers were designed using the Primer Premier 5.0 software based on the sequence differences between the *japonica* rice cultivar Nipponbare and the *indica* cultivar 93-11. DNA was extracted from fresh rice leaves using the CTAB method (Murray and Thompson 1980). PCR-based molecular markers used to identify the genotypes of these progenies are listed in Additional file 2: Table S1. The 20 μl PCR mixture consisted of 50 ng template DNA, 2.0 μl 10× PCR buffer, 0.2 mM dNTP, 0.2 μM of each primer and 0.2 U *Taq* DNA polymerase. The PCR procedure was as follows: initial denaturation step at 94 °C/4 min; followed by 40 cycles of 94 °C/30 s, annealing (temperature changes were based on primers, usually 55 °C) for 30 s, 72 °C/30 s; and a final extension step at 72 °C/10 min. Products were separated with 4 % agarose gels, stained by GelRed and visualized by UV radiation.

### Vector construction and transformation

A 9826-bp wild-type genomic DNA fragment containing the entire *WSL12* coding region, a 2074-bp upstream sequence and a 1641-bp downstream region was amplified by PCR using the primer pair LC1. PCR products were digested with *KpnI* and *XbaI*. The target fragment was purified and then inserted into the binary vector pCAMBIA1300. The recombinant plasmid, named pWSL12 and pCAMBIA1300 (pCK, contained the promoter of *WSL12* and part of the code region), was introduced into the calluses generated from mature seed embryos of *wsl12* mutant using *Agrobacterium*-mediated transformation (Hiei et al. 1994). The 1.9-kb sequence of *WSL12* promoter was amplified by PCR using the primer pair LGUS. PCR products were digested with *EcoRI* and *BglII*. The fragment was purified and then inserted into the binary vector pCAMBIA1305. The recombinant plasmid was used to transform Wuyujing7 following the protocol described above. The positive transgenic plants were visualized using GUS staining assay (Scarpella et al. 2003). For over-expression vector construction, the entire cDNA of *WSL12* was amplified using the primer pair LOE. PCR products were digested with *KpnI* and *XbaI*. The target fragment was inserted into the vector pCAMBIA1300s (35S promoter was inserted into pCAMBIA1300) and then transformed *wsl12*. Primers used for vector construction are listed in Additional file 2: Table S2.

### RNA preparation and quantitative real-time PCR

The total RNA of various samples from *wsl12* and wild-type plants was extracted using the MicroRNA Extraction kit (Axygen, http://www.axygenbio.com), and the first-strand cDNA was synthesized using the ReverTra Ace qPCR RT kit (Toyobo Co. Ltd.; http://www.toyobo.cn/) following the manufacturer’s instructions. The expression levels of *WSL12* and the genes associated with chlorophyll synthesis, chloroplast development, ABA synthesis and abiotic stress were assessed using quantitative real-time PCR (qRT-PCR). *OsActin1* (*Os03g0718100*) was used as the internal control. All relevant primers are listed in Additional file 2: Table S3. QRT-PCR was conducted on Applied Biosystems 7900HT. Each 10 μl reaction system consisted of 1 μl total cDNA, 5 μl 2× SYBR Green PCR Master Mix (TaKaRa Co. Ltd., Otsu, Japan; http://www.takara.com.cn) and 4 μl of 1 μM primers. QRT-PCR conditions were as follows: 95 °C/5 min, followed by 40 cycles of 95 °C/30 s, 60 °C/30 s and 72 °C/30 s. For each sample, qRT-PCR results were measured by four technical replicates and three biological replicates. The 2^−ΔΔCT^ method was adopted to analyze the relative expression levels of relevant genes (Livak and Schmittgen 2001), and two-tailed *t* test was used to detect statistically significant differences.

### Subcellular location of WSL12 in rice protoplast

To investigate the subcellular localization of WSL12 protein, the *WSL12* full-length cDNA without termination codon was cloned into the AHLG (reconstructed from pCAMBIA1300) binary vector. *WSL12* sequence was placed upstream of GFP coding sequence. The fusion plastid and the control plastid (empty vector) were transformed into rice protoplasts according to the protocols described previously (Chen et al. 2006). Fluorescence signals were visualized with confocal fluorescence scanning microscope (LSM780, Carl Zeiss, Germany).

## Results

### Phenotypic characteristics of the *wsl12* mutant

**Fig. 1:**
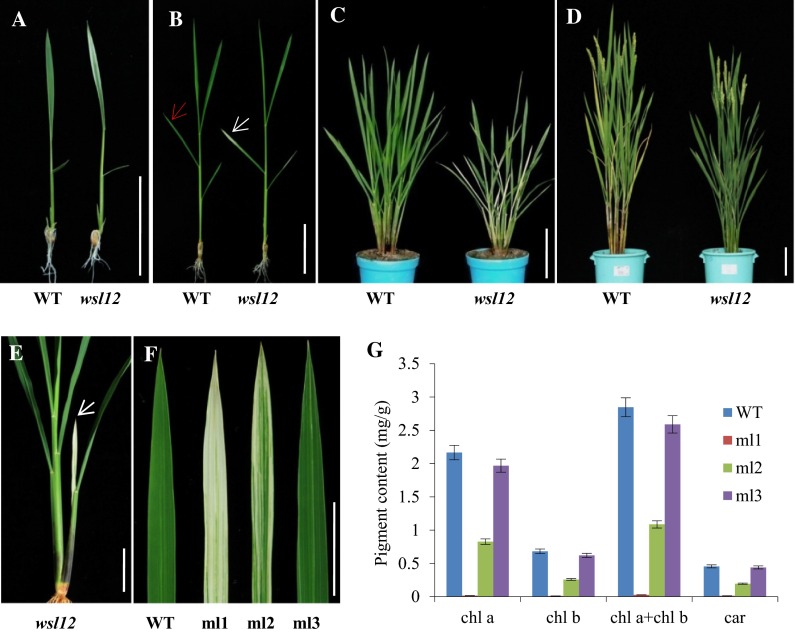
Phenotypic characterization of wild type and the *wsl12* mutant. Phenotypes of wild-type (*left*) and *wsl12* mutant (*right*) plants at two-leaf stage (**a**), four-leaf stage (**b**), tillering stage (**c**) and filling stage (**d**). The *red and white arrows* show normal and white stripe region of leaves in wild type and *wsl12* mutant, respectively. **e** New tiller of *wsl12* mutant, *white arrow* shows the new leaf. **f** Three leaf types of the *wsl12* mutant at tillering stage. **g** Pigment content in leaves of wild-type and *wsl12* mutant at tillering stage. Chlorophyll *a*: chl *a*, chlorophyll *b*: chl *b*, carotenoid: car. Data represent mean ± SD from three independent biological replicates. ml1: *white* leaf in *wsl12*, ml2: *white* stripe leaf in *wsl12*, ml3: *green* leaf in *wsl12. Bars* 5 cm in **a**, **b**, **e** and **f** and 10 cm in **c** and **d**

**Fig. 2:**
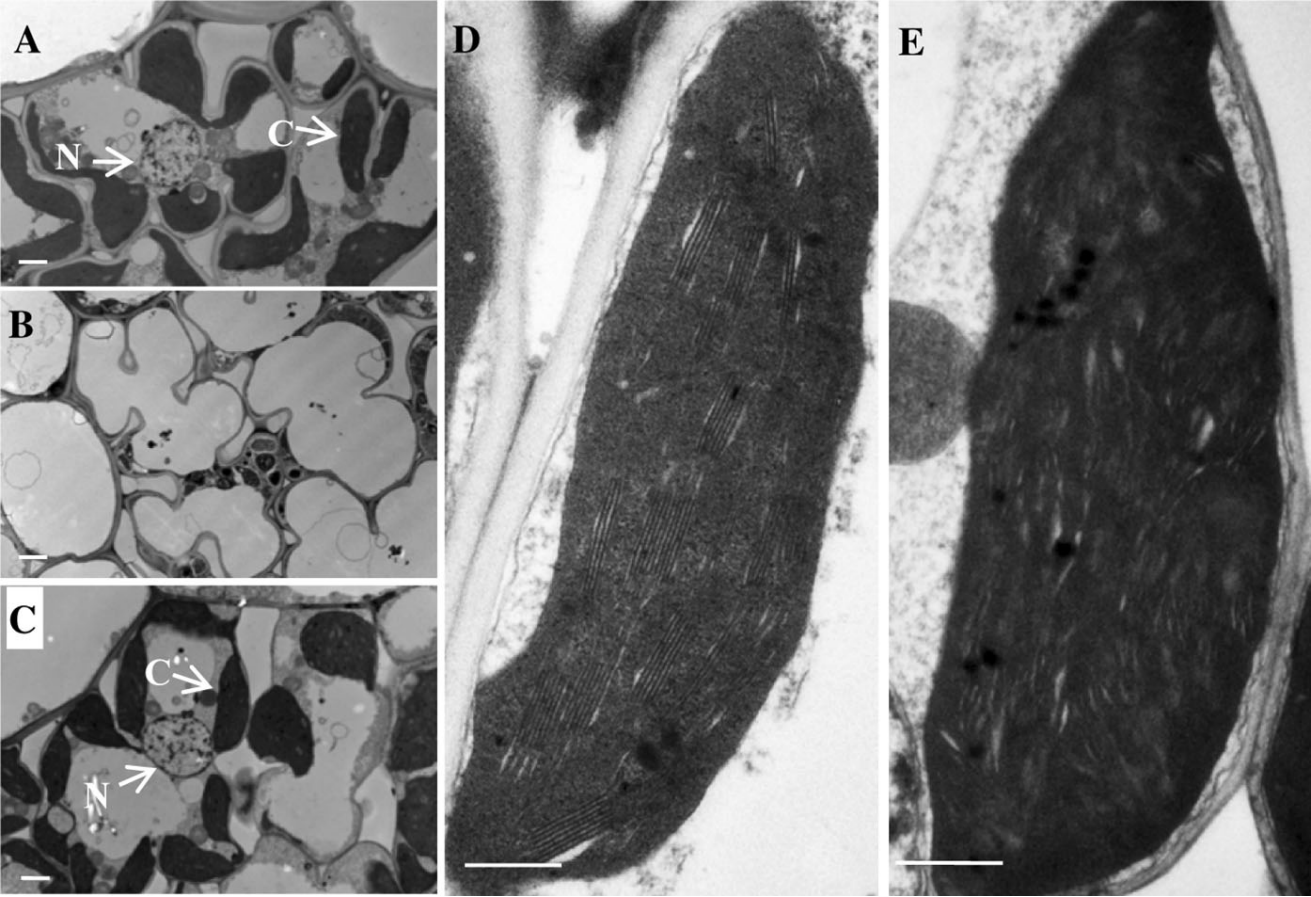
Transmission electron microscopic images of cells from wild type and the *wsl12* mutant at tillering stage. **a** Cells in leaf of wild type. **b** Cells in *white* sectors of leaf in *wsl12* mutant. **c** Cells in *green* sectors of *wsl12* mutant. **d** Chloroplast from wild type. **e** Chloroplast in *green* sectors of the mutant. *Bars* 5 μm in **a**–**c** and 0.5 μm in **d** and **e**. *C* chloroplast, *N* nucleus

The *wsl12* mutant showed white stripe leaf phenotype when the first leaf fully developed (Fig. 1a). But these abnormal leaves gradually reverted to green along with the plant development. The newly development leaves after the third leaf stage displayed green color (Fig. 1b). More interesting, abnormal leaves also appeared at tillering stage. Three types of leaves could be observed in the *wsl12* mutant plants at tillering stage (Fig. 1c, e, f): Almost whole white leaf (ml1) mainly distributed on the new leaves of new tillers (Fig. 1e), white stripe leaf (ml2) and green leaf (ml3) (Fig. 1f). The ml1 leaves gradually turned to green, and then only ml3 leaves could be observed at mature stage (Fig. 1d). The panicles of *wsl12* also showed abnormal phenotype at initial heading stage and then turned to normal. The *wsl12* mutant showed lower photosynthetic rate compared with that of wild-type plants (Additional file 1: Fig. S1). Compared with the wild type, the *wsl12* mutant exhibited significant differences in the major agronomic traits, including plant height, seed-setting rate, heading date and the number of tillers per plant (Additional file 1: Fig. S2). The plant height of *wsl12* was approximately 76.7 ± 1.11 cm, which was 9.25 % shorter than the wild type (83.8 ± 1.1 cm). The seed-setting rate of *wsl12* was about 56.5 ± 3.5 %, which was much lower than that of the wild type (96 ± 0.03 %). The *wsl12* mutant exhibited delayed flowering time and less tillers per plant. There was no significant difference between *wsl12* and wild-type plants in panicle length, the number of primary/secondary branches and 1000-grain weight (Additional file 1: Fig. S2). Previous studies showed that leaf color was closely linked with photosynthetic pigments and chloroplast development (Murchie et al. 2005). Our results indicated that the concentrations of chlorophyll a (Chla), chlorophyll b (Chlb) and carotenoid in ml1 and ml2 leaves decreased significantly compared with that in the wild type, whereas the pigment concentrations of ml3 leaves were almost normal (Fig. 1g). Transmission electron microscopy analysis showed the lamellar structure of chloroplasts in the wild type was well developed (Fig. 2a, d). But in the *wsl12* mutant, there was no chloroplast or chloroplast-like organelle in ml1 leaves (Fig. 2b). Although the chloroplast number and size between ml3 and wild type did not show significant difference (Fig. 2c), there was no well-organized lamellar structure in the ml3 leaves (Fig. 2e). These observations indicate that the *wsl12* phenotypes are closely associated with chloroplast development and chlorophyll synthesis.

### The phenotype of *wsl12* is affected by temperature

To examine whether the pigment content in the mutant *wsl12* is affected by temperature, we compared the pigment concentrations between the *wsl12* mutant and wild-type plants grown at the constant temperature of 22, 26, 30, and 34 °C conditions, respectively. At 22 °C condition, the leaves of *wsl12* mutant exhibited severe albino phenotype at three-leaf stage (Additional file 1: Fig. S3a), and a large decrease in pigment concentrations compared with that of wild-type plants (Additional file 1: Fig. S3e). When the seedlings were exposed to 26 °C condition, *wsl12* mutant showed much lower albino degree and pigment concentrations increased (Additional file 1: Fig. S3b, f). When seedlings were grown at 30 °C condition, there was almost no difference between *wsl12* mutant and wild-type plants (Additional file 1: Fig. S3c, g). Interestingly, we could easily detect their difference at 34 °C (Additional file 1: Fig. S3d, i). These results demonstrate that the *wsl12* phenotype is strongly affected by temperature.

### The single-base deletion of *WSL12* is responsible for the *wsl12* phenotype

To determine whether the *wsl12* phenotype is controlled by a single gene, we performed genetic analysis of reciprocal crosses between *wsl12* and the *japonica* cultivars Wuyujing7. The results showed that *wsl12* was a recessive mutant, based on examination of individuals of the F_2_ segregating population. The ratio of individuals with normal phenotype to mutant phenotype was 3:1 (χ^2^ = 0.358 < χ^2 0.05,1^ = 3.84), indicating that the *wsl12* phenotype was controlled by a single recessive nuclear gene.

**Fig. 3:**
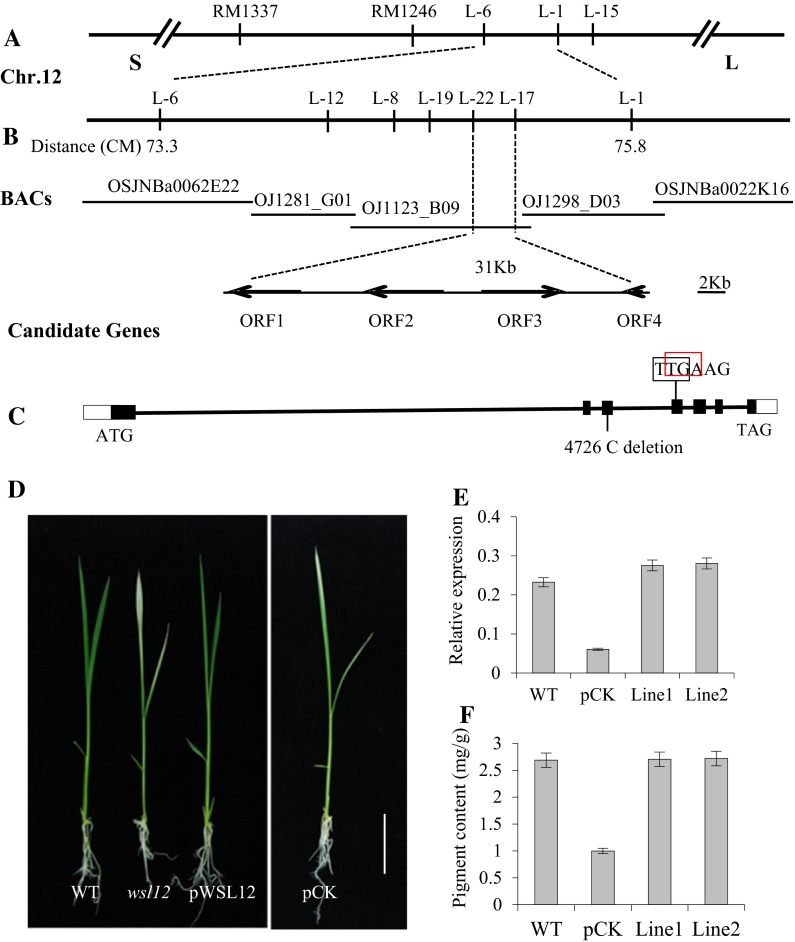
Map-based cloning of *WSL12* and transgenic complementation of the *wsl12* mutant. **a** *WSL12* was initially mapped to a 2.5-cM interval on chromosome 12 between markers L-6 and L-1. **b** *WSL12* locus was narrowed to a 31-kb region between markers L-22 and L-17 on BAC clone OJ1123_B09. Four open reading frames (ORFs) were predicted in the mapped region. **c** Structure of *WSL12* (ORF3), ATG, TGA represent for start and stop codon, *black boxes* indicate exons, and *lines between black boxes* indicate introns. *Black pane* shows the right translation way, one single nucleotide deletion result in premature transcription termination (*red* pane). **d** Phenotypes of wild-type, *wsl12*, transgenic-positive plants and transgenic plants carried negative controlled vector (pCK). *Scale bar* 2 cm. **e** Expression analysis of *WSL12* in young leaves of wild-type, pCK, and two complemented lines (*Line1*, *Line2*) by qRT-PCR. **f** Total pigment content of young leaves from wild-type, pCK, and two complemented lines. Data represent mean ± SD based on three independent biological experiments

We constructed the mapping population by crossing the *wsl12* mutant with the *indica* cultivar 93-11. Bulked segregant analysis (BSA) was used to produce a primary map of *WSL12*. PCR genotyping was carried out using a bulk DNA pool from 33 *wsl12*/93-11 F_2_ individuals with the mutant phenotype, and 118 molecular markers scattered on all of the rice chromosomes were used to determine the approximate map position of *WSL12*. *WSL12* was primarily located on the long arm of chromosome 12, closely linked to SSR marker RM1246 (Fig. 3a). To further fine map *WSL12*, we designed new markers next to RM1246 based on the sequence difference between *japonica* rice variety Nipponbare and *indica* variety 93-11 (http://www.gramene.org/resources/). The polymorphism primers were subsequently used to screen the genotypes of 2, 257 individuals with mutant phenotype and then mapped *WSL12* between two markers, L-22 and L-17, within a 31-kb physical interval (Fig. 3b). There were four predicted ORFs according to the Rice Genome Annotation Project (http://rice.plantbiology.msu.edu/index.shtml; Fig. 3b). Sequence analysis revealed only a single-base deletion in the third ORF (*LOC_Os12g36194*, *OsNDPK2*) of the *wsl12* mutant. Bioinformatic analysis showed that *WSL12* consisted of 6,111 nucleotides, contained seven exons (Fig. 3c) and encoded a 331 amino acid protein. Compared with the wild type, the deletion was in the third exon and caused a frameshift mutation which resulted in premature translation termination (Fig. 3c, Addition file 1: Fig. S4). Genetic complementation verified the identity of the *WSL12* candidate gene. We introduced the complementary plastid containing the entire *OsNDPK2* ORF, and pCK vector as a negative control, into the *wsl12* mutant. The phenotype of *wsl12* was restored to normal in the positive transgenic plants (Fig. 3d), whereas all pCK lines failed to recover the wild-type phenotype, which demonstrated that the *wsl12* phenotype can specifically rescue by introducing the *OsNDPK2* genomic fragment (Fig. 3d, e). In addition, we detected the pigment content of wild-type and complementary plastid transgenic plants and found that the pigment content in the wild-type and positive transgenic plants was higher than that of pCK transgenic lines (Fig. 3f). Moreover, over-expressing *WSL12* in *wsl12* mutant plants showed that the mutant phenotype could be restored to normal phenotype (Addition file 1: Fig. S5). These results suggest that *LOC_Os12g36194* is responsible for the *wsl1*2 phenotype.

### Expression pattern of *WSL12*

**Fig. 4:**
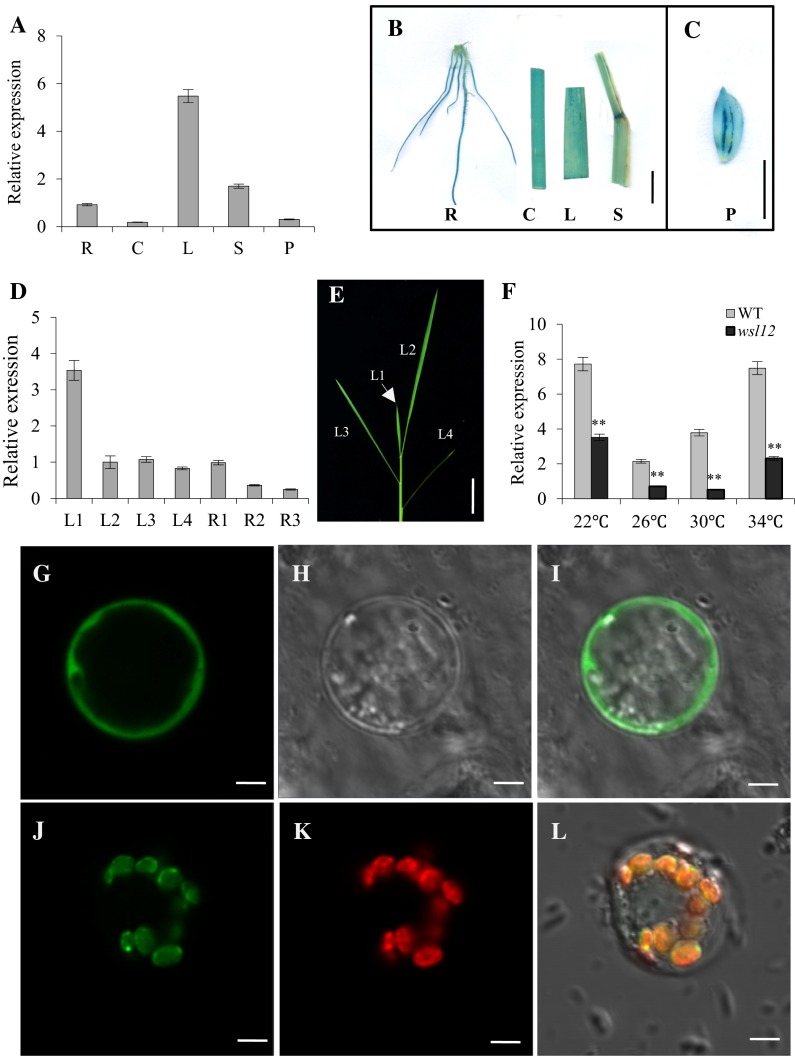
Expression pattern analysis and subcellular localization of *WSL12*. **a** QRT-PCR estimation of the relative expression level of *WSL12*. RNA was isolated from root (R), culm (C), leaf blade (L), leaf sheath (S) and young panicle (P) of wild-type plants. **b–c** *WSL12* expression was revealed by β-glucuronidase (GUS) staining in *WSL12* promoter-GUS transgenic plants. **d** QRT-PCR analysis of relative expression levels of *WSL12* in leaves and roots at different development stages. R1, R2 and R3 represent roots of wild-type at 3, 7 and 30 days after germination, respectively. **e** Main leaves of 4-week-old wild-type plants grown at Hangzhou field conditions. **f** Expression levels of *WSL12* in leaves of wild-type plants and the mutant under temperature treatment. *Bars* 1 cm in **b** and **e** and 5 mm in **c**. Data represent mean ± SD based on three independent biological replicates, and *asterisk* indicates a significant difference (Student’s *t* test, *P < 0.05; **P < 0.01). Rice protoplasts cell expressing empty GFP vector. **g** GFP florescence. **h** Bright-field image of GFP. **i** Merged image of **g** and **h**. Rice protoplasts cell expressing the WSL12-GFP fusion protein. **j** GFP florescence. **k** Auto-fluorescence of chloroplasts. **l** Merged image of **j** and **k**. *Scale bar* = 5 μm

Quantitative real-time PCR (qRT-PCR) was used to analyze the expression pattern of *WSL12*. We examined the expression levels of *WSL12* transcripts in root, culm, leaf blade, leaf sheath and immature panicle. The expression level of *WSL12* was higher in leaves (Fig. 4a). β-glucuronidase (GUS) staining showed the same results as that of qRT-PCR. GUS staining signals appeared in the entire tissues in normal field conditions (Fig. 4b, c), which indicated that *WSL12* displayed as constitutive expression pattern in rice. Since the albino phenotype was more obvious in newly developed leaves at seedling and tillering stages (Fig. 1a, e), we detected the expression levels of *WLS12* between new and old tissues. Our results demonstrated that *WSL12* was highly expressed in young tissues (Fig. 4d). We also examined the expression levels of *WSL12* in the leaves of plants grown under different temperatures. We found that the expression levels of *WSL12* were higher in plants grown at 22 and 34 °C than those grown at 26 and 30 °C (Fig. 4f). These result indicated that *WSL12* may be responsible for the temperature stress.

### Chloroplast localization protein WSL12 regulates the expression of the genes associated with chlorophyll synthesis and chloroplast development

To examine the subcellular location of WSL12, we constructed the WSL12-GFP fusion vector and introduced it into rice protoplast cells. The green fluorescent signals of WSL12-GFP were colocalized with the auto-fluorescent signals of chloroplasts, suggesting that WSL12 located in the chloroplasts (Fig. 4g–l). The expression levels of genes associated with chlorophyll synthesis and chloroplast development determine pigment content and chloroplast developmental status. The expression patterns of these genes were examined in *wsl12* and wild-type plants under normal field conditions. They were *glutamyl*-*tRNA reductase* (*HEMA1*), *Mg*-*chelatase subunit H* (*CHLH*), *3, 8*-*divinyl protochlorophyllide a 8*-*vinyl reductase* (*DVR*), *pchlide oxidoreductase* (*PORA*), *chlorophyllide a oxygenase* (*CAO1*), *chlorophyll synthase* (*YGL1*), *small subunit of rubisco* (*rbcS*), *light*-*harvesting Chl a/b*-*binding protein of PSII*, *photosynthesis*-*related* (*cab1R*) and *cab2R*. The expression levels of these genes were significantly decreased in the *wsl12* plants (Additional file 1: Fig. S6). The other genes related to photosynthesis including *psaA*, *psbA* (encoding two reaction center polypeptides) and *rbcL* (encoding the large subunit of rubisco) were also down-regulated in the *wsl12* plants. But *rpoA* and *rpoB* (two genes related to RNA polymerase) were significantly up-regulated in the *wsl12* plants (Additional file 1: Fig. S6). These results demonstrate that *WSL12* plays an important role in regulating the expression of genes associated with chlorophyll biosynthesis, chloroplast development and photosynthesis.

### The *wsl12* mutant is sensitive to ABA and salinity stresses

**Fig. 5:**
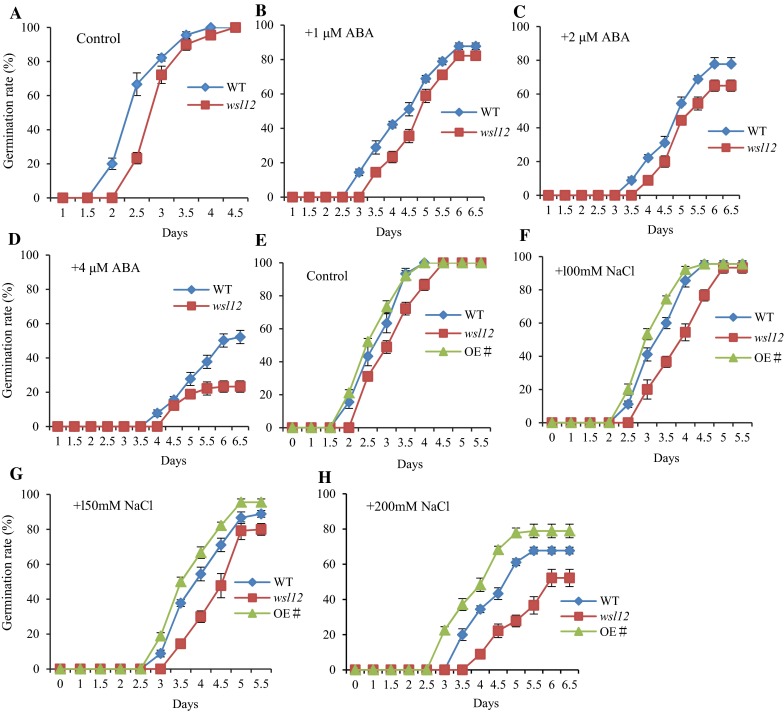
Effect of ABA and NaCl on the growth of plants. Germination rate of wild-type and *wsl12* seeds in the absence (**a**) and presence of 1 μM (**b**), 2 μM (**c**) or 4 μM (**d**) of ABA. Germination rate of wild-type, *wsl12* and over-expression seeds in the absence (**e**) and presence of 100 mM (**f**), 150 mM (**g**) or 200 mM (**h**) of NaCl. The germination was identified as the coleoptiles were grown at least 5 mm long. Data represent mean ± SD based on three independent biological replicates, and *asterisk* indicates a statistically significant difference (Student’s *t* test, *P < 0.05; **P < 0.01). The *X* axis means germination rate, and *Y* axis represents days

Under normal field conditions, we found that there was significant difference between *wsl12* and wild-type plants in the germination time. The *wsl12* mutant seeds germinated later than wild-type seeds. In previous studies, abnormal ABA concentrations that can affect seed germination have been reported. We detected the ABA concentration in *wsl12* and wild-type plants. The ABA concentration of *wsl12* mutant decreased obviously compared with wild-type plants (Additional file 1: Fig. S7a). The transcription levels of the genes related to ABA synthesis were decreased in the *wsl12* mutant (Additional file 1: Fig. S7b). Therefore, we speculated that *WSL12* might regulate ABA response in rice. Seed germination tests showed that the germination rate of *wsl12* mutant was lower than that of wild-type plants with exogenous 2 μM ABA (Fig. 5c). High concentration of ABA (4 μM) could inhibit the germination of wild-type (50 % germination rate) and mutant seeds (20 % germination rate), while the inhibition was much stronger in *wsl12* seeds (Fig. 5d). Besides, ABA-induced inhibition on shoot and root length was stronger in *wsl12* mutant compared with wild-type plants when exogenous ABA concentration increased from 2 to 4 μM (Additional file 1: Fig. S7c–e). These observations demonstrate that the *wsl12* mutant is sensitive to ABA-mediated inhibition at the seed germination and seedling stage (Fig. 5a–d; Additional file 1: Fig. S7). Thus, *WSL12* can significantly affect ABA response and biosynthesis.

Since salinity response is closely associated with ABA in plant (Tan et al. 2014), we examined the salinity response in *wsl12* mutant and *WSL12* over-expression plants by adding excessive NaCl to the 1/2 MS medium. The *wsl12* mutant showed delayed seed germination, but there was no significant difference between wild-type and *WSL12* over-expression plants in the germination time without additional NaCl (Fig. 5e). The germination time of *WSL12* over-expression plants was earlier than that of wild-type plants and *wsl12* mutant with 200 mM NaCl (Fig. 5h). However, the germination rate of *wsl12* mutant decreased with excessive NaCl (Fig. 5f–h). In contrast, the germination rate of *WSL12* over-expression plants was higher than the wild-type at 200 mM NaCl condition (Fig. 5h). These results demonstrate that *wsl12* mutant is more sensitive to NaCl.

### The superoxide level is high in *wsl12* mutant

ABA is an important hormone regulating plant growth and development. Previous studies showed that ABA can enhance the tolerance of plants against stress through inducing the expression of antioxidant enzymes including superoxide dismutase (SOD), CAT and ascorbate peroxidase (APX; Kaminaka et al. 1999; Anderson et al. 1994). Thus, we investigated the expression levels of these genes and oxidative status of cells in the leaves of *wsl12* mutant and wild-type plants at seedling stage. Nitrotetrazolium blue chloride (NBT) staining showed that the oxidative status in *wsl12* leaves was more severe than that of wild type (Additional file 1: Fig. S8a). The expression levels of *APX1*, *APX2* and *CatA* were down-regulated, while the transcription levels of *AOX1a* and *SODA1* were up-regulated in *wsl12* mutant (Additional file 1: Fig. S8b). These results indicate that *WSL12* participates in reactive oxygen species-scavenging pathway by regulating the expression of these genes. AtNDPK2 can directly interact with phytochrome A since it acts as a positive signal transducer in the phytochrome signaling pathway (Choi et al. 1999). Thus, we detected the expression levels of three phytochrome genes (*PHYA*, *PHYB* and *PHYC*) and the other two *OsNDPKs* (*OsNDPK1* and *OsNDPK3*). We found that the expression levels of the three phytochrome genes and *OsNDPK1* were down-regulated in *wsl12* mutant (Additional file 1: Fig. S8c). However, the expression level of *OsNDPK3* which involved in the signaling for stress response was significantly increased in *wsl12* mutant (Escobar Galvis et al. 2001; Liu et al. 2015), which might compensate for the function of *OsNDPK2*.

## Discussions

The diverse phenotypes in rice mutants affecting chlorophyll synthesis and chloroplast development are characterized by seedling viability and/or leaf coloration, such as virescent (*v*), stripe (*st*), albino, chlorina, zebra and yellow-variegated leaves (Jung et al. 2003). *V1* encodes a chloroplast-located protein NUS1 which is essential for establishing rice plastid genetic system during early development (Kusumi et al. 2011). *V2* encodes a guanylate kinase located in both plastid and mitochondria and functions in chloroplast differentiation (Sugimoto et al. 2004, 2007). *V3* and *ST1* encode the large and small subunits of ribonucleotide reductase, respectively. They are mainly involved in DNA synthesis and repair during early leaf development (Yoo et al. 2009). *WSL* encodes a pentatricopeptide repeat protein which is required for the accumulation of plastids in ribosomes and affects the splice of chloroplast *rpl2* transcripts (Tan et al. 2014). The *wsl*, *v1*, *v2*, *v3* and *str1* mutants are all sensitive to temperature. The phenotypes of *wsl* plants are more obvious in low temperature (Tan et al. 2014). The leaves of *v1* and *v2* mutants become chlorotic when grown under restrictive temperatures (≤20 °C), but show nearly deep green color at the temperature ≥30 °C. On the contrary, *v3* and *st1* produce bleached leaves at the constant temperature of 20 or 30 °C, but generate almost green leaves under the temperature between 20 and 30 °C (Kusumi et al. 2011; Sugimoto et al. 2007; Yoo et al. 2009). The *wsl12* mutant, also a temperature-sensitive mutant, showed conspicuous abnormal leaves under the constant temperature of 22 or 34 °C. However, at the temperature of 30 °C, the leaves of *wsl12* mutant were almost as green as wild-type plants (Additional file 1: Fig. S3c). These results show that the phenotype of *wsl12* mutant is strongly affected by temperature. We can observe white stripe leaves in newly developed leaves of *wsl12* mutant (Fig. 1a, e). The seed-setting rate of *wsl12* mutant was significantly decreased, which may due to weakened photosynthesis caused by the abnormal chloroplast structure (Fig. 2, Additional file 1: Fig. S1). The panicles of *wsl12* mutant also showed white-green phenotype at initial heading stage, demonstrating that *WSL12* also affects the chloroplast development in parenchyma cells of palea and lemma.

Map-based cloning and complementary experiment demonstrate that *WSL12* encodes OsNDPK2. *WSL12* expressed in various tissues and highly expressed in leaves and young tissues (Fig. 4a–d), which can explain that the albino phenotype of new leaves in the *wsl12* mutant was more obvious. Contradictory results have been reported on the cellular location of NDPK2. Although many data and results showed that NDPK2 should be targeted to the chloroplast (Yang and Lamppa 1996; Bovet et al. 1999), AtNDPK2 was falsely considered to have nuclear and cytoplasmic locations in *Arabidopsis* (Zimmermann et al. 1999; Choi et al. 1999). Later reports thoroughly revisited the location of AtNDPK2 and demonstrated that AtNDPK2 was exclusively targeted to chloroplasts (Bölter et al. 2007; Jaedicke et al. 2011). They consider that previous unreasonable results may be caused by the use of inadequate constructs for detecting the location of AtNDPK2. In this study, the fluorescent signals of WSL12-GFP were colocalized with the auto-fluorescent signals of chloroplasts, which confirmed that OsNDPK2 targeted to chloroplasts (Fig. 4g–l). The expression levels of genes associated with chlorophyll biosynthesis and chloroplast development were down-regulated, except for *rpoA* and *rpoB* (Additional file 1: Fig. S6), which indicated that *WSL12* was required for the normal transcription of these genes. The expression pattern of *rpoA* and *rpoB* was different from other chlorophyll biosynthetic genes, but the mechanism that caused the difference needs to be further researched. In addition, the mutation of *wsl12* repressed the transcription of genes involved in photosynthesis, such as *psaA*, *psbA*, *rbcL* and *rbcS*, which may be the underlying mechanism of the low photosynthetic rate in *wsl12*.

Various environmental stresses, including extreme temperature, salt, drought, excessive light and osmotic shock, can cause high ROS levels. ROS is an important cellular regulator for stress response and oxidative cell death (Moon et al. 2003). Chloroplasts, the major cellular component for photosynthesis, are highly exposed to ROS damage since photosynthesis is a source of ROS and H_2_O_2_. Therefore, chloroplast is also involved in triggering ROS/redox-dependent signaling (Petrov and Van Breusegem 2012; Kangasjärvi et al. 2012). Many evidences have confirmed that *AtNDPK2* is involved in ROS signaling and oxidative stress management (Yang et al. 2003). Over-expressing *AtNDPK2* induces the expression of multiple antioxidant genes, which can enhance the resistance to oxidative stress (Yang et al. 2003; Kim et al. 2009). This protective role against oxidative stress makes *NDPK2* a useful target for plant biotechnology. Over-expression *AtNDPK2* in plants can enhance tolerance against multiple stresses (Kim et al. 2009; Moon et al. 2003). In our study, over-expressing *OsNDPK2* could enhance rice germination rate under salt stress (Fig. 5), which may be a useful germplasm resource with high tolerance against environment stress such as salinity for rice molecular breeding.

In recent years, the area of arable land is decreased because of environment pollution. With the increasing saline-alkali soil and drought land, food production faces the risk of reduction. Some experts concerned that global warming also threatened grain output. Previous studies and this work proved that *NDPK2* is involved in oxidative stress regulation and G protein regulation pathway (Shen et al. 2008). Some G protein-related genes in rice have been cloned. For example, a key enhancing yield gene *DEP1* or nutrient-efficient gene *DEP1*-*1* has been identified and participates in G protein activity. It can provide a breeding strategy to improve new varieties for environmentally sustainable increases in nitrogen use efficiency and rice grain yield (Sun et al. 2014). These findings provide an important genetic information for improving crop yield and abiotic stress tolerance via molecular breeding application of *NDPK2* gene (Guo and Ye 2014; Qian et al. 2016).

The *wsl12* mutant showed higher superoxide anion level compared with the wild-type plants (Additional file 1: Fig. S8a). But not all of the genes related to anti-oxidate were down-regulated (Additional file 1: Fig. S8b). We speculated that other genes, such as *OsNDPK3* which was highly expressed in *wsl12* mutant, might involve in the regulation of cellular redox state. We observed conspicuous phenotypic difference between *wsl12* and wild-type plants at the temperature of 22 and 34 °C, but not at 30 °C (Additional file 1: Fig. S3). The expression level of *WSL12* in wild-type plants under extreme temperature was higher than that under mild temperature, which is consistent with the function of *WSL12* since temperature stress could cause high ROS levels. ROS could accumulate in the cells of *wsl12* mutant, suggesting that these cells may partially dead due to the loss function of *WSL12* (Fig. 2b).

Previous reports demonstrate that AtNDPK2 is an upstream component of the phytochrome-mediated light signaling pathway in *Arabidopsis thaliana* (Choi et al. 1999; Shen et al. 2005). In *wsl12* mutant, three phytochrome genes (*PHYA*, *PHYB* and *PHYC*) were all down-regulated (Additional file 1: Fig. S8c). Previous studies show that phytochrome B regulates chlorophyll synthesis and chloroplast development and also affects ABA metabolism (Zhao et al. 2012; Gu et al. 2012). Thus, further studies were necessary to confirm whether the expression change in the genes related to chlorophyll synthesis was directly caused by the loss function of *WSL12*. Actually, the ABA concentration of *wsl12* mutant decreased obviously compared with wild-type plants (Additional file 1: Fig. S7a), but the mutant plants were more sensitive to ABA-induced inhibition (Fig. 5a–d; Additional file 1: Fig. S7c-e). This may because exogenous ABA can induce hydrogen peroxide (H_2_O_2_) and superoxide radical (O_2_^−^) production (Pei et al. 2000; Jiang and Zhang 2001), but the *wsl12* mutant had lower ROS tolerance because of the loss function of *WSL12*. This demonstrated that the extent of high ROS level repressing plants growth was more serious in *wsl12* than in wild-type plants. In addition, auxin and ROS are interconnected in a complex signaling network regulating plant growth and development (Tognetti et al. 2012). It is possible that the phenotype of *wsl12* mutant is affected by auxin signaling and ROS pathways. In summary, all these discoveries suggest that WSL12 is a multifunctional protein involved in chlorophyll synthesis, cell signaling and stress responses. Further studies of WSL12 should focus on elucidating the complex network among ROS, phytochrome-mediated and auxin-mediated signal transduction pathways.

## Electronic supplementary material

Below is the link to the electronic supplementary material.

Supplementary material 1 (DOCX 2326 kb)

Supplementary material 2 (DOCX 22 kb)
