# Supplementary material for: White stripe leaf 12 (WSL12), encoding a nucleoside diphosphate kinase 2 (OsNDPK2), regulates chloroplast development and abiotic stress response in rice (Oryza sativa L.)
Source: Mol Breed. 2016 Apr 29;36:57. doi: 10.1007/s11032-016-0479-6 (PMC4851688; doi:10.1007/s11032-016-0479-6)
Supplement: Supplementary file 1 — Supplementary material 1 (DOCX 2326 kb) [file 11032_2016_479_MOESM1_ESM.docx]

**B**


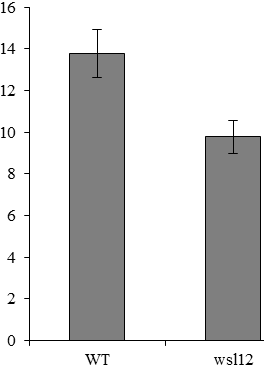


**

WT *wsl12*


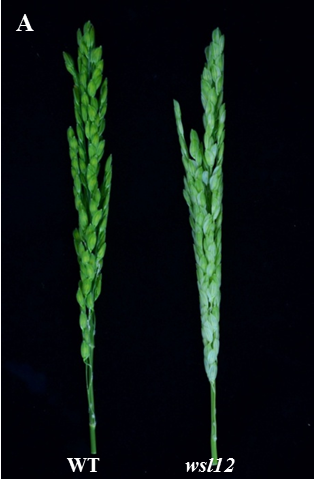


Photosynthetic rate (μmol CO_2_ m^-2^ s^-1^)

**Figure S1** Panicle phenotype and photosynthetic rate of wild-type and *wsl12.* **a** Panicle phenotype of wild-type (left) and *wsl12* mutant (right) plant at initial heading stage at Hangzhou field conditions. **b** Photosynthetic rate of wild-type and *wsl12.* Data represent mean ± SD based on data from five plants, asterisk indicates significant difference (Student’s t-test, ** P < 0.01).

**E**


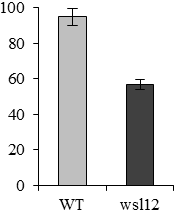


Seed-setting rate/%

**


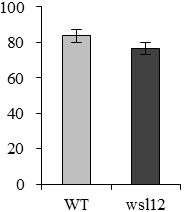


Plant height/cm

**

**A**


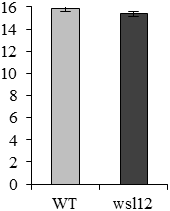


Panicle length/cm

**B**


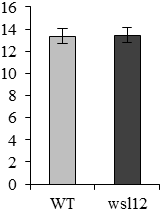


No. of primary branches

**C**


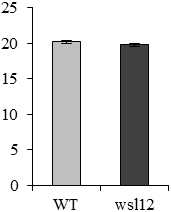


No. of secondary branches

**D**


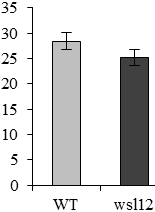


1000 grains weight/g

**F**


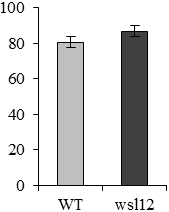


Heading date/day

*

**G**


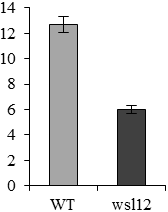


No. of tillers per plant

**

**H**

**Figure S2** Agronomic traits performance of wild-type and *wsl12* plants. **a** Plant height. **b** Panicle length. **c** No. of primary branches. **d** No. of secondary branches. **e** Seed-setting rate. **f** 1000 grains weight. **g** Heading date. **h** Tillers per plant. Data represent mean ± SD based on three independent biological replicates, and asterisk indicates a significant difference (Student’s t-test, * P < 0.05; ** P < 0.01).


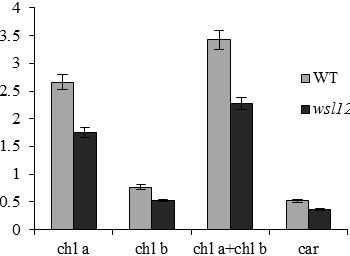


**

**

**

*

pigment content (mg/g)

**F**

26 ℃


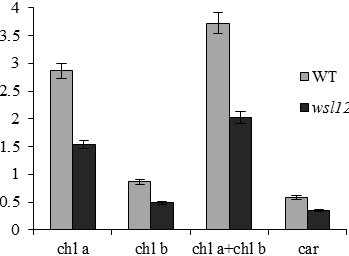


**H**

**

**

**

*

34 ℃

pigment content (mg/g)


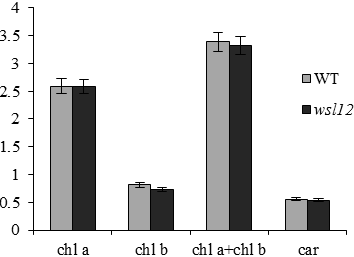


**G**

30 ℃

pigment content (mg/g)

**E**

pigment content (mg/g)


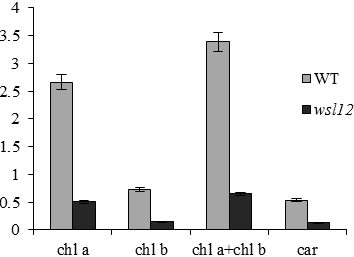


22 ℃

**

**

**

*


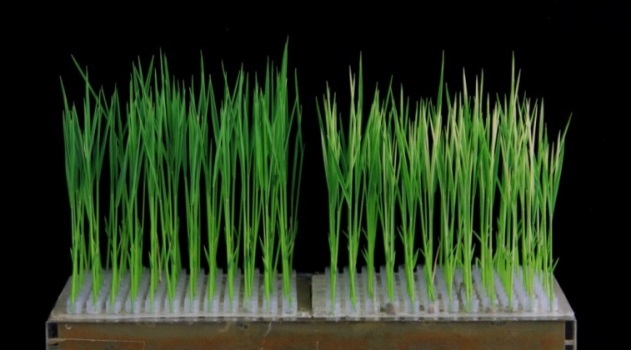

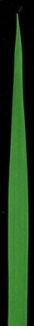

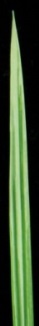


**D 34℃**


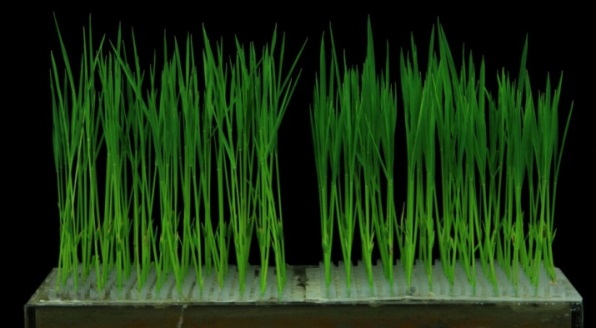

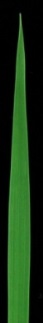

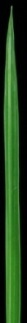


**C 30℃**

**WT *wsl12* WT *wsl12***


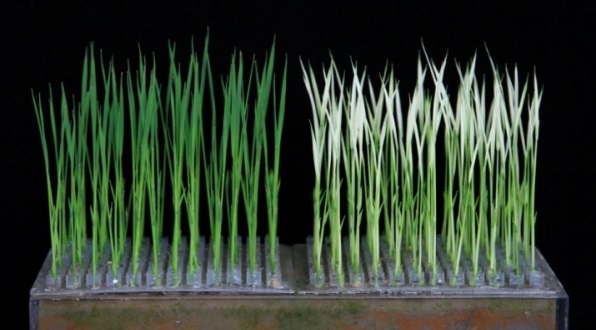

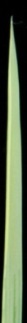

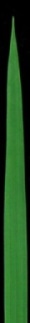


**A 22℃**


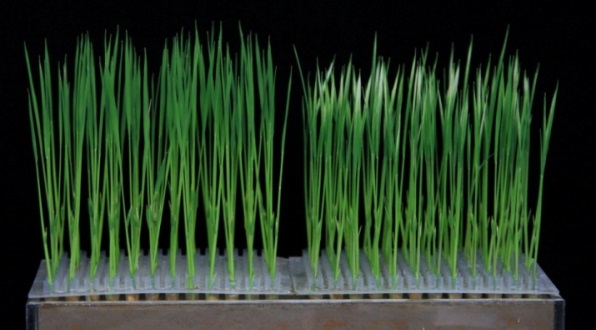

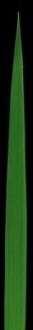

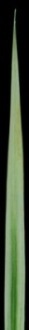


**B 26℃**

**WT *wsl12* WT *wsl12***

**Figure S3** Phenotypes and pigment content of leaves from wild-type and mutant under temperature treatment. **a-d** Phenotypes of wild-type and the *wsl12* plants under 22 ℃ (**a**), 26 ℃ (**b**), 30 ℃ (**c**), and 34 ℃ (**d**) conditions. Scale bar=2.5 cm. **e**-**h** Pigment content in leaves of wild-type and *wsl12* mutant plants at three-leaf stage under 22 ℃ (**e**), 26 ℃ (**f**), 30℃ (**g**), and 34℃ (**h**) conditions in the growth chamber. Data are shown as mean ± SD of three independent assays. Asterisk indicates a significant difference (Student’s t-test, * P < 0.05; ** P < 0.01).

**
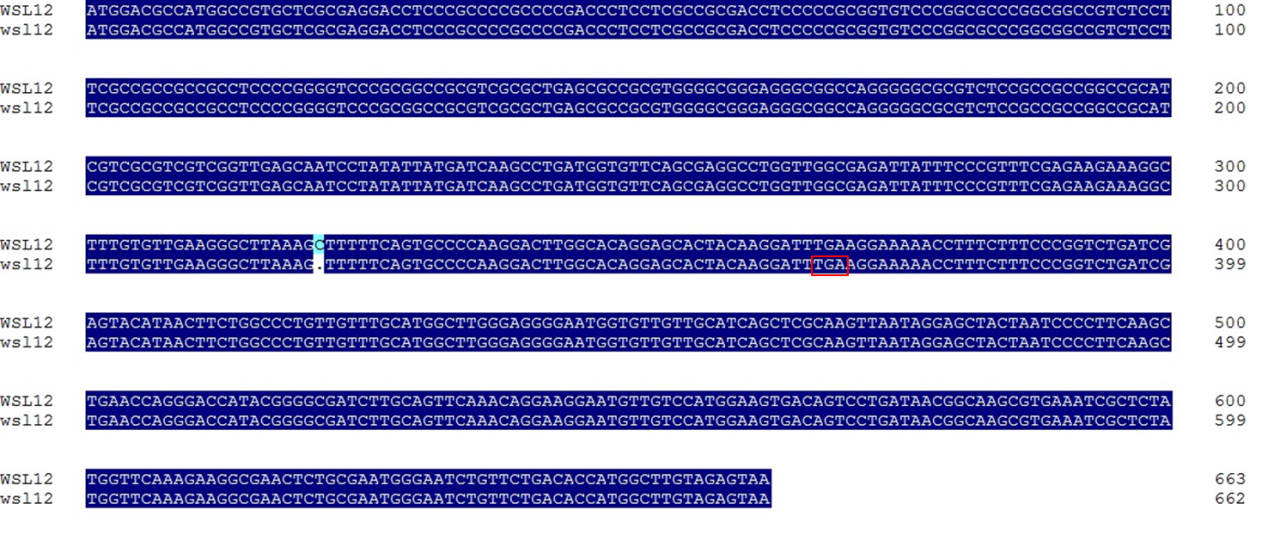
**

**Figure S4** Comparison of the cDNA sequence of WSL12 between the mutant and wild-type. One base deletion caused a frameshift mutation which resulted in premature translation termination in the mutant.

**
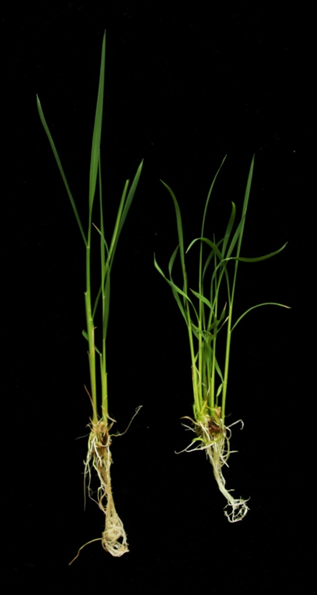
**

**Line1 Line2**

**Figure S5** Phenotype of over-expressing *WSL12* in *wsl12* mutants. Scale bar = 2cm.


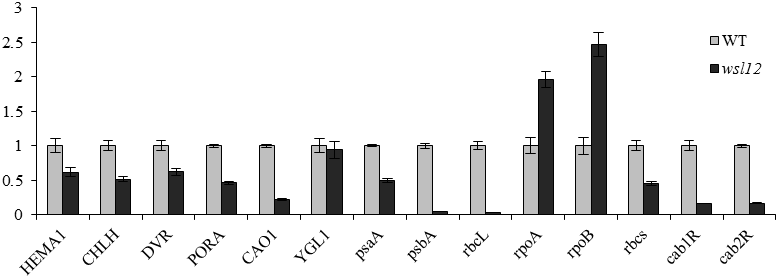


Relative expression

**Figure S6** Expression analysis of genes associated with chlorophyll biosynthesis, chloroplast development and photosynthesis by qRT–PCR. Total RNA was isolated from leaves of wild-type and white strip sectors of *wsl12* at three-leaf stage under Hangzhou field conditions. Data represent mean ± SD based on three independent biological experiments.

**A**


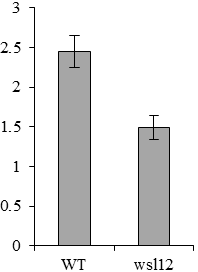


ABA concentration (ng/g)

**

**B**


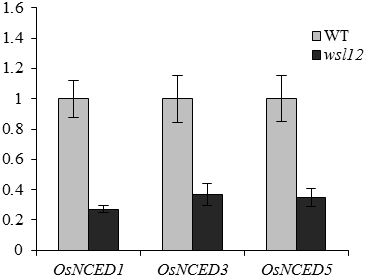


**

**

**

Relative expression

ABA (μM) 0 1 2 4


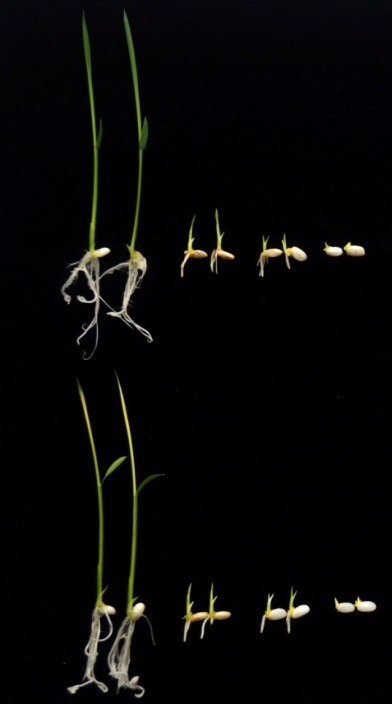


**WT**

***wsl12***

**C**


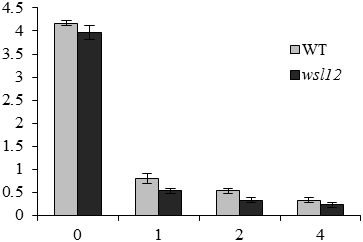


Root length (cm)

ABA (μM)

*

*

**E**


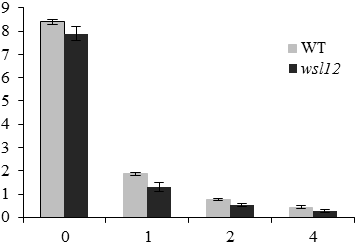


Shoot length (cm)

**

**

**D**

ABA (μM)

**Figure S7** **a** ABA concentration in leaves of wild-type and *wsl12*. **b** Expression levels of genes related to ABA synthesis. RNA was isolated from leaves at four-leaf stage. **c** Phenotypes of seven-day-old wild-type and wsl12 seedlings on media supplemented with increasing concentrations of ABA, Bar = 2 cm. **d-e** Shoot length (**d**) and root length(**e**) of wild-type and *wsl12* seedlings in (**c**). Data represent mean ± SD based on three independent biological replicates, and asterisk indicates a statistically significant difference (Student’s t-test, * P < 0.05; ** P < 0.01).

**B**


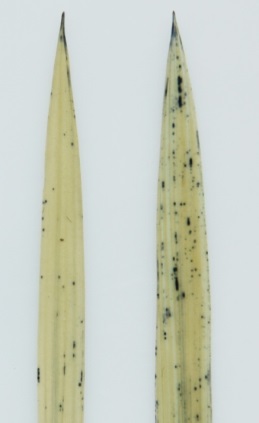


**WT *wsl12***

**A**


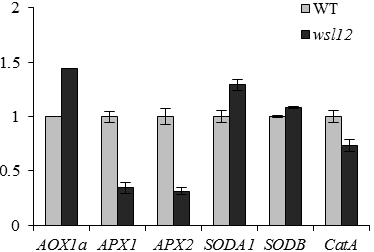


**

**

**

**

**

Relative expression

**

**C**


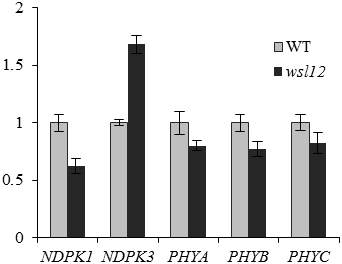


**

**

**

Relative expression

*

**

**Figure S8** Detection of superoxide anion and relative expression levels of genes associated with ROS detoxification*,* nucleoside diphosphate kinase and phytochrome in leaves of wild-type and *wsl12*. **a** Detection of superoxide anion in leaves of wild-type and the *wsl12* mutant was carried out by NBT staining. **b** Expression analysis of genes associated with ROS detoxification. **c** Expression analysis of *NDPK1*, *NDPK3*, *PHYA*, *PHYB*, and *PHYC* in wild-type and the *wsl12* mutant. Total RNA was extracted from leaves of wild-type and *wsl12* at three-leaf stage under paddy field conditions. Data represent mean ± SD based on three independent biological replicates, and asterisk indicates a significant difference (Student’s t-test, * P<0.05; ** P < 0.01).
