# Supplementary material for: White stripe leaf 12 (WSL12), encoding a nucleoside diphosphate kinase 2 (OsNDPK2), regulates chloroplast development and abiotic stress response in rice (Oryza sativa L.)
Source: Mol Breed. 2016 Apr 29;36:57. doi: 10.1007/s11032-016-0479-6 (PMC4851688; doi:10.1007/s11032-016-0479-6)
Supplement: Supplementary file 2 — Supplementary material 2 (DOCX 22 kb) [file 11032_2016_479_MOESM2_ESM.docx]

**Table S1** Primers used for *WSL12* mapping

| Markers | Sense (5’–3’) | Anti-sense (5’-3’) | | Product size (bp) |
| --- | --- | --- | --- | --- |
| RM1337 | GTATCCTTTCTCGCAATCGC | | GTGGCCCGAACCTGTATAAC | 125 |
| RM1246 | AGCTCGATCCCCTAGCTCTC | | TTGGAGAAGGTCACCTGCC | 188 |
| L-1 | GTGTTATCGACCCCTTTG | | TCATGGCACCTATCAGCT | 96 |
| L-6 | TTGATGACGAGCGTAGTTG | | AGTGATGACCGATGAAGA | 243 |
| L-8 | TGGCTGGACCATTAGAAA | | TTTTCCCTAATGTGTCCACGTC | 187 |
| L-12 | GCTTCCTAACCGATAAAA | | TTTGACTCCCAATCCTAT | 156 |
| L-15 | ATCAGGATCTATGAACTTGCA | | ATCAGCTACACTTTGGACTCA | 84 |
| L-17 | CTACCACGATCAAGATGC | | GGGCTCTCCAATTTATCT | 219 |
| L-19 | CCTCCATACAGATGAAGTT | | GATAGGTTGAGCAGGTTA | 170 |
| L-22 | ATCCACTATGGCACTACGC | | AGATCACTGACATGTTCACCT | 170 |

**Table S2** Vector construction primers

| Markers | Primer pairs (5’-3’) | Restriction enzyme |
| --- | --- | --- |
| LC1-F | CCTTGAGGTACCGCGTTGCTGTATAGAAAGCG | KpnI |
| LC1-R | AATCTAGAATGTCTTGCTTGCAATTCCTATCAGGCAGG | XbaI |
| LGUS-F | CCGGAATTCCTTGAGGTACCGCGTTGCTGTATAGAAAG | EcorI |
| LGUS-R | GAAGATCTACCATCCCGGTAGCTGCTTCTCCTCCAACGTCC | BglII |
| LGFP-F | TAGGGCCCATGGACGCCATGGCCGTGCTCGCGAGGA | ApaI |
| LGFP-R | GCTCTAGACTCTACAAGCCATGGTGTCAGAACAG | XbaI |
| LOE-F | CGGGGTACCATGGACGCCATGGCCGTGCTCGCGAGG | KpnI |
| LOE-R | GCTCTAGATTACTCTACAAGCCATGGTGTCAGAACAG | XbaI |

**Table S3** Quantitative real-time PCR primers used in this study

| Marker | | Sense (5’–3’) | Anti-sense (5’–3’) | Reference |
| --- | --- | --- | --- | --- |
| *HEMA1* | CGCTATTTCTGATGCTATGGGT | | TCTTGGGTGATGATTGTTTGG | Song et al.2013 |
| *CHLH* | CTATACATTCGCCACACT | | TATCACACAACTCCCAAG | Tan et al. 2014 |
| *DVR* | CGAGCCCAGGTTCATCAAGGTGC | | CCTCCCGATCTTGCCGAACTCC | Song et al.2013 |
| *PORA* | TGTACTGGAGCTGGAACAACAA | | GAGCACAGCAAAATCCTAGACG | Tan et al. 2014 |
| *CAO* | GATCCATACCCGATCGACAT | | CGAGAGACATCCGGTAGAGC | Song et al.2013 |
| *YGL1* | AACCTTACCGTCCTATTCCTT | | CCATACATCTAACAGAGCACCC | Song et al.2013 |
| *PsaA* | GCGAGCAAATAAAACACCTTTC | | GTACCAGCTTAACGTGGGGAG | Song et al.2013 |
| *PsbA* | CCCTCATTAGCAGATTCGTTTT | | ATGATTGTATTCCAGGCAGAGC | Song et al.2013 |
| *rbcL* | CTTGGCAGCATTCCGAGTAA | | ACAACGGGCTCGATGTGATA | Song et al.2013 |
| *rpoA* | GTGGAAGTGTGTTGAATCAA | | TCTCTCTTGATCCGTAACTC | Song et al.2013 |
| *rpoB* | TTTGGTTTCGATGTGCA | | TATGGTCTAATTCCGAGCGGT | Song et al.2013 |
| *rbcs* | TCCGCTGAGTTTTGGCTATTT | | GGACTTGAGCCCTGGAAGG | Song et al.2013 |
| *CAB1R* | AGATGGGTTTAGTGCGACGAG | | TTTGGGATCGAGGGAGTATTT | Song et al.2013 |
| *CAB2R* | TGTTCTCCATGTTCGGCTTCT | | GCTACGGTCCCCACTTCACT | Song et al.2013 |
| *AOX1a* | CTTCGCATCGGACATCCATTA | | TCCTCGGCAGTAGACAAACATC | Tan et al. 2014 |
| *APX1* | AGGTGCCACAAGGAAAGATCTGGT | | TCAGCAGGGCTTTGTCACTAGGAA | Tan et al. 2014 |
| *APX2* | TGGGAAGATGCCACAAGGAGAGAT | | TCCGCAGCATATTTCTCCACCAGT | Tan et al. 2014 |
| *SODA1* | ATCTGGATGGGTGTGGCTAGCTTT | | AGTACGCATGCTCCCAGACATCAA | Tan et al. 2014 |
| *SODB* | TCCGCCGTATAAACTTGATGCCCT | | TGGGTTGCCGTTGTTGTATGCTTC | Tan et al. 2014 |
| *catA* | CAACCGCAACGTCGACAACTTCTT | | TTCACCGGCAGCATCAGGTAGTTT | Tan et al. 2014 |
| *PHYA* | CACATACTTACCGTTGTAGAGGA | | CACAGGCATTCACAACCAAGAT |  |
| *PHYB* | AAGGAATCTGAGGAAACAGTAAAC | | CCCGAATGTCTTCAACTTTATCTC |  |
| *PHYC* | CGATGATTCTGTTGAAGTGGTT | | GGTCCATTATTTTCCTGGTGATT |  |
| *NDPK1* | AGAAGGAGATCGCTCTCTGG | | AGATCCAAGGGTGAAGGTTG |  |
| *WSL12* | CTTTTTCAGTGCCCCAAGGA | | TCGATCAGACCGGGAAAGAA |  |
| *NDPK3* | ATTGGTGCTACAGACCCACA | | CTCAGCCTTTGCAGTCTCTG |  |
| *OsActin* | TGGCATCTCTCAGCACATTCC | | TGCACAATGGATGGGTCAGA |  |
